# Compressive Strength Estimation of Fly Ash/Slag Based Green Concrete by Deploying Artificial Intelligence Models

**DOI:** 10.3390/ma15103722

**Published:** 2022-05-23

**Authors:** Kaffayatullah Khan, Babatunde Abiodun Salami, Mudassir Iqbal, Muhammad Nasir Amin, Fahim Ahmed, Fazal E. Jalal

**Affiliations:** 1 Department of Civil and Environmental Engineering, College of Engineering, King Faisal University (KFU), P.O. Box 380, Al-Hofuf, Al-Ahsa 31982, Saudi Arabia; mgadir@kfu.edu.sa; 2 Interdisciplinary Research Center for Construction and Building Materials, King Fahd University of Petroleum and Minerals, Dhahran 31261, Saudi Arabia; salami@kfupm.edu.sa; 3 Shanghai Key Laboratory for Digital Maintenance of Buildings and Infrastructure, State Key Laboratory of Ocean Engineering, School of Naval Architecture, Ocean & Civil Engineering, Shanghai Jiao Tong University, Shanghai 200240, China; mudassiriqbal29@sjtu.edu.cn (M.I.); jalal2412@sjtu.edu.cn (F.E.J.); 4 Department of Civil Engineering, University of Engineering and Technology, Peshawar 25120, Pakistan; 5 Department of Physics, College of Science, King Faisal University, P.O. Box 380, Al-Hofuf, Al-Ahsa 31982, Saudi Arabia; fahmed@kfu.edu.sa

**Keywords:** compressive strength, blast furnace slag, fly ash, green concrete, artificial intelligence, GEP, ANFIS, GBT

## Abstract

Cement production is one of the major sources of decomposition of carbonates leading to the emission of carbon dioxide. Researchers have proven that incorporating industrial wastes is of paramount significance for producing green concrete due to the benefits of reducing cement production. The compressive strength of concrete is an imperative parameter to consider when designing concrete structures. Considering high prediction capabilities, artificial intelligence models are widely used to estimate the compressive strength of concrete mixtures. A variety of artificial intelligence models have been developed in the literature; however, evaluation of the modeling procedure and accuracy of the existing models suggests developing such models that manifest the detailed evaluation of setting parameters on the performance of models and enhance the accuracy compared to the existing models. In this study, the computational capabilities of the adaptive neurofuzzy inference system (ANFIS), gene expression programming (GEP), and gradient boosting tree (GBT) were employed to investigate the optimum ratio of ground-granulated blast furnace slag (GGBFS) and fly ash (FA) to the binder content. The training process of GEP modeling revealed 200 chromosomes, 5 genes, and 12 head sizes as the best hyperparameters. Similarly, ANFIS hybrid subclustering modeling with aspect ratios of 0.5, 0.1, 7, and 150; learning rate; maximal depth; and number of trees yielded the best performance in the GBT model. The accuracy of the developed models suggests that the GBT model is superior to the GEP, ANFIS, and other models that exist in the literature. The trained models were validated using 40% of the experimental data along with parametric and sensitivity analysis as second level validation. The GBT model yielded correlation coefficient (R), mean absolute error (MAE), and root mean square error (RMSE), equaling 0.95, 3.07 MPa, and 4.80 MPa for training, whereas, for validation, these values were recorded as 0.95, 3.16 MPa, and 4.85 MPa, respectively. The sensitivity analysis revealed that the aging of the concrete was the most influential parameter, followed by the addition of GGBFS. The effect of the contributing parameters was observed, as corroborated in the literature.

## 1. Introduction

Concrete is the most abundant and widely used manufactured material on our planet. It is a remarkable material and technological advancements constantly alter its attributes and applications in our built environment. The global annual concrete production is approximately 30 billion tons [1]. Concrete has incredible characteristics such as its durability, strength, versatility, and affordability, making it an ideal building material for meeting societal goals in terms of sustainable development, affordable housing, and resilient infrastructure [2]. Compared with other building materials, the embodied energy and carbon footprint of concrete is very low [3]. However, because of its wide use in a variety of applications, its enormous production results in a substantial carbon footprint, contributing to 8% of global carbon dioxide emissions [4]. Cement, a major concrete component, accounts for only 12% of its weight but is responsible for 95% of its carbon footprint. Cement is one of the largest contributors to embodied carbon in the built environment, generating 7% of the world’s CO_2_ emissions [5]. Owing to the anticipated rise in population and future challenges of urbanization, cement demand is projected to grow by 12 to 23% by 2050 [6]. Climate change has become more prevalent during the past few decades, and scientists have attributed much of this to anthropogenic carbon dioxide emissions and global warming [7]. Carbon dioxide emissions contribute to climate change and global warming. The demand for low-carbon concrete increases daily in response to climate change mitigation and adaptation [8]. Hence, it is vital to look for pathways to reduce emissions in the cement and concrete industry to reduce its environmental impact [5].

Several technologies have been proposed to reduce concrete’s environmental impact in recent years, including clinker reduction, carbon capture, fuel switching, energy efficiency improvements, etc. [9]. Clinker reduction by replacing it with supplementary cementitious materials is the most effective, practical, and easily applicable to ready-mix concrete industry [10]. Different potential supplementary cementitious materials such as fine limestone (L) [11] fly ash (FA) [12], ground granulated blast furnace slag (GGBFS) [13], silica fume (SF) [14], volcanic ash [15] (VA), calcined clay (CC) [16], rice husk ash (RHA) [17], bagasse ash (BA) [18], waste glass sludge (WGS) [19], and nanosilica (NS) [20], among others, have been investigated for use as a potential substitute to clinker for the production of sustainable cement and concrete. In recent decades, granulated blast furnace slag (GBFS), a byproduct of the iron industry, and fly ash (FA), a byproduct of thermal coal plants, have been the most common clinker substitutes. Currently, cement contains about 20% SCMs that substitute Portland cement clinker—predominantly fine limestone, GGBFS, and FA. Approximately 330 of FA and 900 Mt/yr. of GBFS are available globally [16]. Many studies have been conducted to study the effect of different percentage replacements of cement with FA and GGBFS, and its effect on mechanical and durability properties. According to previous research [21], concrete made by incorporating FA and GGBFS shows better mechanical properties and durability than control concrete, particularly at later ages. The compressive strength is an important mechanical characteristic of concrete and correlated nonlinearly with the percentage composition of constituent materials, which becomes further complicated with the addition of green material [22]. To avoid costly laboratory testing and improve the efficacy of engineering projects, it is desirable to develop an intelligent model that can accurately capture nonlinear behavior of attributes in yielding the compressive strength of green concrete containing FA and GGBFS, which would achieve an optimized mixture to produce strong and sustainable concrete for attaining targeted sustainability goals in the construction industry.

Over the last two decades, machine learning (ML) algorithms, namely artificial neural networks (ANN), gene expression programming (GEP), multiexpression programming (MEP), decision trees (DT), gradient boosting tree (GBT), among others, have been widely used to predict the mechanical properties of concrete owing to their capabilities of advanced and extremely powerful yielding of solutions to nonlinear and complex problems [23]. Sadowski et al. [24] investigated the compressive strength of green concrete prepared by partially replacing cement with waste quartz dust employing nonlinear capabilities of ANN by deploying 156 samples. Using 103 experimental values, Singh et al. [25] investigated green concrete incorporated with FA and GGBFS using ANN and obtained a correlation coefficient of 0.89 in predicting the compressive strength. Bui et al. [26] successfully improved ANN with a modified firefly algorithm (ANN-MFA) to estimate the compressive strength of FA- and GGBFS-based high-performance green concrete. Zarandi et al. [27] investigated the capabilities of fuzzy polynomial neural networks (FPN) on 458 experimental samples to develop various prediction models for silica-fume- and fly-ash-incorporated green concrete based on variable setting parameters, achieving an accuracy of correlation equaling 0.82 for the best model. Similarly, Chou et al. [28] obtained a correlation of 0.91 for predicting FA- and GGBFS-incorporated green concrete. Chou et al. [29] developed support vector regression (SVR), chi-square automatic interaction detector (CHAID), linear regression (LR), and ANN models for estimating the compressive strength of concrete incorporating a variety of SCMs. Several other researchers employed the tree-based M5P model [30], ANN modified with wavelet gradient boosted, bagging, gradient boosting and wavelet boosting [31], linear regression (LR), and genetic operation trees (GOT) [32], among others, for the compressive strength of green concrete, achieving lowest MAE and RMSE of 3.36 and 4.51; however, the accuracy of these models in terms of error analysis suggests the development of more accurate models. Additionally, these models estimate the compressive strength similar to a black box, furnishing no prediction formulas for designers and practitioners.

Moreover, the literature mentioned above emphasized the application of machine learning techniques rather than the actual interpretation of the future recommendations regarding the optimum content of FA and GGBFS together with the water-to-binder ratio. In addition, the effect of variable setting parameters on the performance of the models is not clearly described [33]. Mousavi et al. [34] developed a model based on gene expression programming (GEP) to estimate the compressive strength of FA and GGBFS incorporated concrete; however, the detailed performance with respect to variable genetic parameters was not reported. This led to a lower accuracy obtained for the developed GEP model. Therefore, this study investigated the capability of variable setting parameters of the GEP model to achieve the best hyperparameters, and used the important significance of the GEP model to furnish simple mathematical relations for predicting the compressive strength of green concrete.

An adaptive neurofuzzy inference system has strong nonlinear capabilities of prediction, combining the advantages of fuzzy logic and ANN [35]. However, the prediction performance of ANFIS regarding FA and GGBFS has been reported very scarcely [36]. Moreover, gradient boosting trees (GBT) has been reported to be a more accurate model among ANN, neural expert system (NES), FPN, multiple additive regression tree (MART), WGB-ANN, random forest (RF), and ANN cross-validation techniques [33]; therefore, the current study employed GBT to forecast the compressive strength of FA- and GGBFS-incorporated concrete. In summary, a comprehensive study is presented here to evaluate the effect of genetic variables, namely, the number of chromosomes, number of genes, and head size, on the performance of the developed models. Moreover, the current study also evaluated the capability of the adaptive neurofuzzy inference system (ANFIS) to estimate a similar problem. Finally, an accurate model in the form of a gradient boosting tree (GBT) is presented to estimate the compressive strength of OPC-based green concrete containing FA and GGBFS. The remainder of the paper is organized as follows: the Research Methodology section presents the details concerning the datasets and the adopted ML algorithms used to train them. The Results and Discussion section describes the results, and the final section presents the research conclusions of this study.

## 2. Research Methodology

This section describes the experimental dataset used to develop the ANFIS, GEP, and GBT models, followed by a rationalization of the variables considered in this study. Herein, a brief overview of the development of the proposed models is presented.

### 2.1. Experimental Database

**Figure 1:**
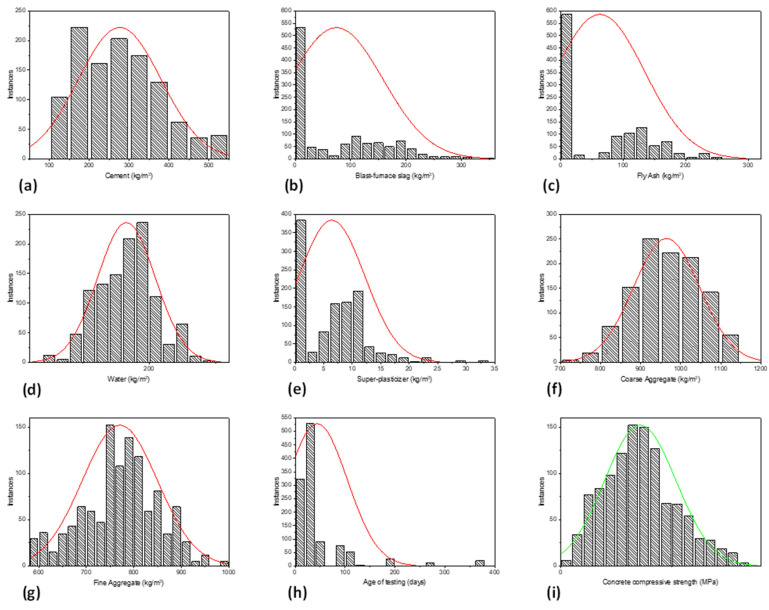
Distribution histogram of varaibles. (**a**) Cement, (**b**) Blast furnace slag, (**c**) Fly Ash, (**d**) water, (**e**) superplasticizer, (**f**) Coarse Aggregates, (**g**) Fina Aggregates, (**h**) Age of testing, (**i**) Concrete compressive strength; Red line shows normal distribution curve for inputs; Green line denotes normal distribution curve for the output variable.

In order to establish a robust AI model, a database of cleaned data comprising 1133 FA- and GGBFS-incorporated concrete test results was gathered and formulated, previously compiled by Nguyen et al. [37], was utilized for training the three algorithms considered in this study. The dataset is for green concrete mixtures with cement, blast furnace slag, fly ash, water, superplasticizer, coarse aggregate, fine aggregate, and age of testing as the input factors and concrete compressive strength (CS) the output factor. The parameters (experimental design variables) employed in this study are listed in Table 1. Figure 1a–h depicts the distribution histograms of the input and output parameters (Figure 1i) used while developing models that applied fuzzy, genetic, and ensemble machine learning approaches. These plots help identify various parameter values that require more data. As the aforementioned input parameters are interdependent, the Pearson correlation coefficients (represented by r) among these variables were determined and are presented in Table 2.

A detailed investigation of the influences of the input parameters revealed that the amount of cement (r = 0.49), superplasticizer (r = 0.35), and age of testing (r = 0.32) were strongly positive, whereas the water, fine aggregate, and coarse aggregate exhibited negative correlations (r = 0.28, 0.16, and 0.15, respectively) with the output parameter. Hence, it can be stated that evaluating the impact of the aforementioned input factors on the compression strength of concrete is convenient and robust.

### 2.2. Prediction Modeling

#### 2.2.1. Adaptive Neurofuzzy Inference System (ANFIS) Model

**Figure 2:**
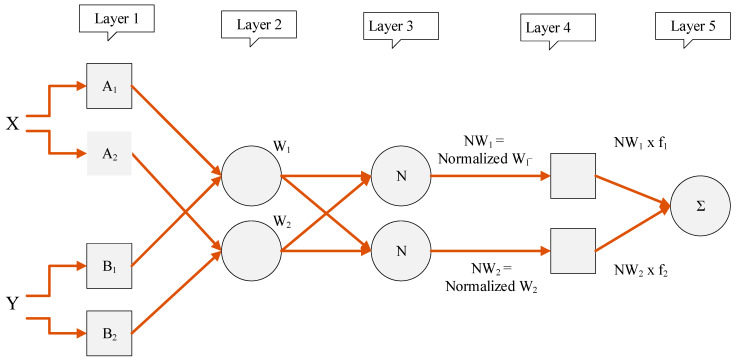
Typical architecture of the ANFIS algorithm.

ANFIS is a hybrid learning technique that combines the learning rules of neural networks with fuzzy logic [38]. The fuzzy inference system of ANFIS corresponds to its fuzzy if–then rules that can be used to learn the problem in the form of a nonlinear function [39]. Figure 2 shows the typical five-layered architecture of the ANFIS algorithm with two inputs and one output variable. In this study, eight input variables were used to predict the compressive strength of the green concrete. Each input variable has two membership functions that carry the degree of satisfaction for each input with respect to the quantifier. The nodes in the second layer calculate the firing strength of the rules. The next layer was responsible for normalizing the firing strength. The subsequent layer defuzzifies the firing strength to calculate the sum of all the incoming signals in the final combining layer. Table 3 shows the hyperparameters obtained during modeling using the ANFIS. A Sugeno-type FIS was used by employing the subclustering method for FIS generation. The training was executed in a hybrid manner to incorporate back-propagation to optimize the error. The optimum FIS was generated at an aspect ratio of 0.5 with 100 iterations.

#### 2.2.2. Gene Expression Programming (GEP) Model

**Figure 3:**
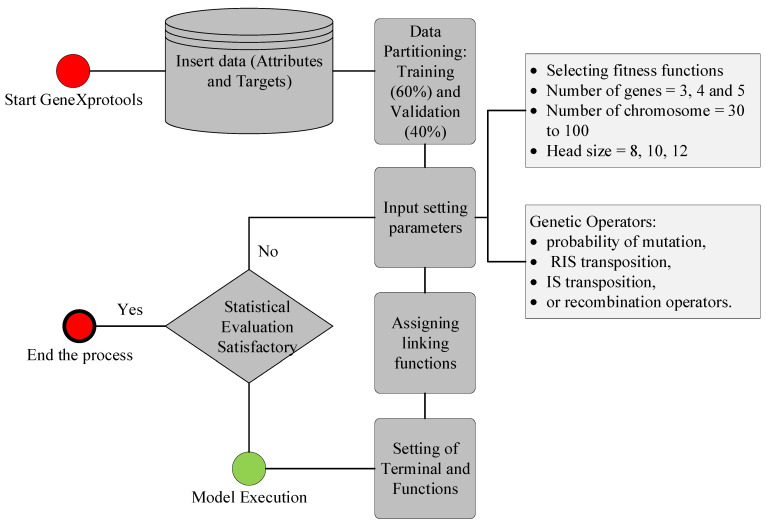
Operation of GEP modeling using GeneXprotools.

The GEP algorithm, developed by Ferreira [40], is an advanced version of genetic algorithms and genetic programming that employs the capabilities of both algorithms. According to Mitchell [41], GEP uses the population of the algorithm and employs its fitness for selection purposes alongside introducing genetic operators to create genetic variation. The attractiveness of the GEP algorithm lies in its ability to forecast the results in the form of simple mathematical relationships in terms of input and output variables [42]. In this study, the GEP algorithm was used to generate a mathematical equation for the compressive strength of green concrete. To initiate the algorithm, GEP creates a random number of populations based on the given number of genes, chromosomes, head size, linking functions, and genetic operators. The schematics encompassing GEP modeling are presented in Figure 3. Initially, data were fed into the interface of GeneXprotools, assigning attributes as targets and predictors. The data were randomly divided into training and validation data, based on the appropriate percentages. The input setting parameters, such as fitness function, number of genes, chromosomes, and head size, were set to 30, 3, and 8, respectively, to initiate the problem. Similarly, genetic operators were determined according to previous literature, as presented in Figure 3. The linking functions for the different genes were assigned according to the previous literature shown in Table 4. The model was executed, and the performance of the model was assessed using assigned fitness functions such as correlation coefficient (R), root mean square error (RMSE), mean absolute error (MAE), and relative squared error (RSE). The mathematical equations for these evaluation functions are given in Equations (1)–(4). The model was executed until there was no significant convergence in maximizing the correlation values or minimizing the magnitude of errors.

**Figure 4:**
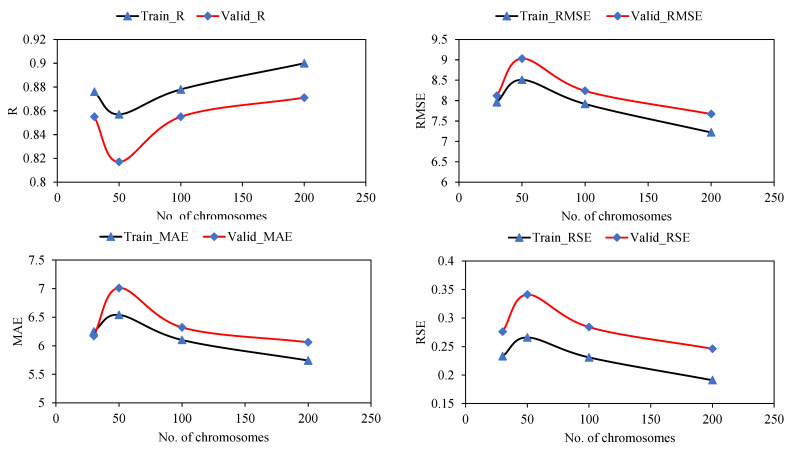
Effect of number of chromosomes on statistical evaluation of the developed GEP models.

According to previous studies, setting the best parameters for the GEP model depends on trial and error [43]. As listed in Table 5, various trials were conducted to determine the best hyperparameters of the GEP algorithm for this particular problem. In an effort to find the best hyperparameters, the effect of various genetic variables, such as the number of chromosomes, number of genes, and head size, was also examined. Figure 4 shows the performance of the models with changing numbers of chromosomes for the training and validation datasets. As a consequence of the change in the number of chromosomes from 30 to 50, the value of R and the corresponding errors, namely, RMSE, MAE, and RSE, increased for both the training and validation data. However, further increases in chromosomes from 50 to 200 considerably increased the correlations. The corresponding magnitude of the errors decreased significantly when the number of chromosomes increased from 50 to 200.

**Figure 5:**
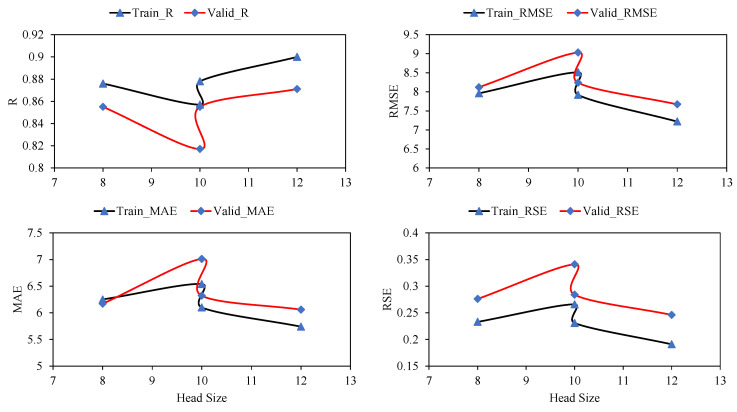
Effect of head size on statistical evaluation of the developed GEP models.

**Figure 6:**
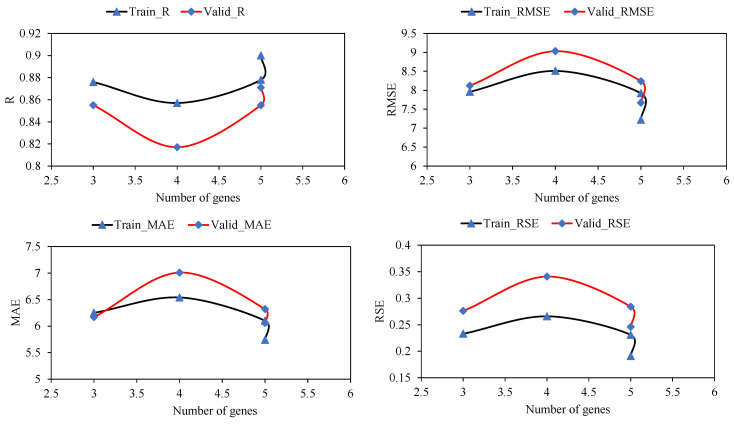
Effect of number of genes on statistical evaluation of the developed GEP models.

**Figure 7:**
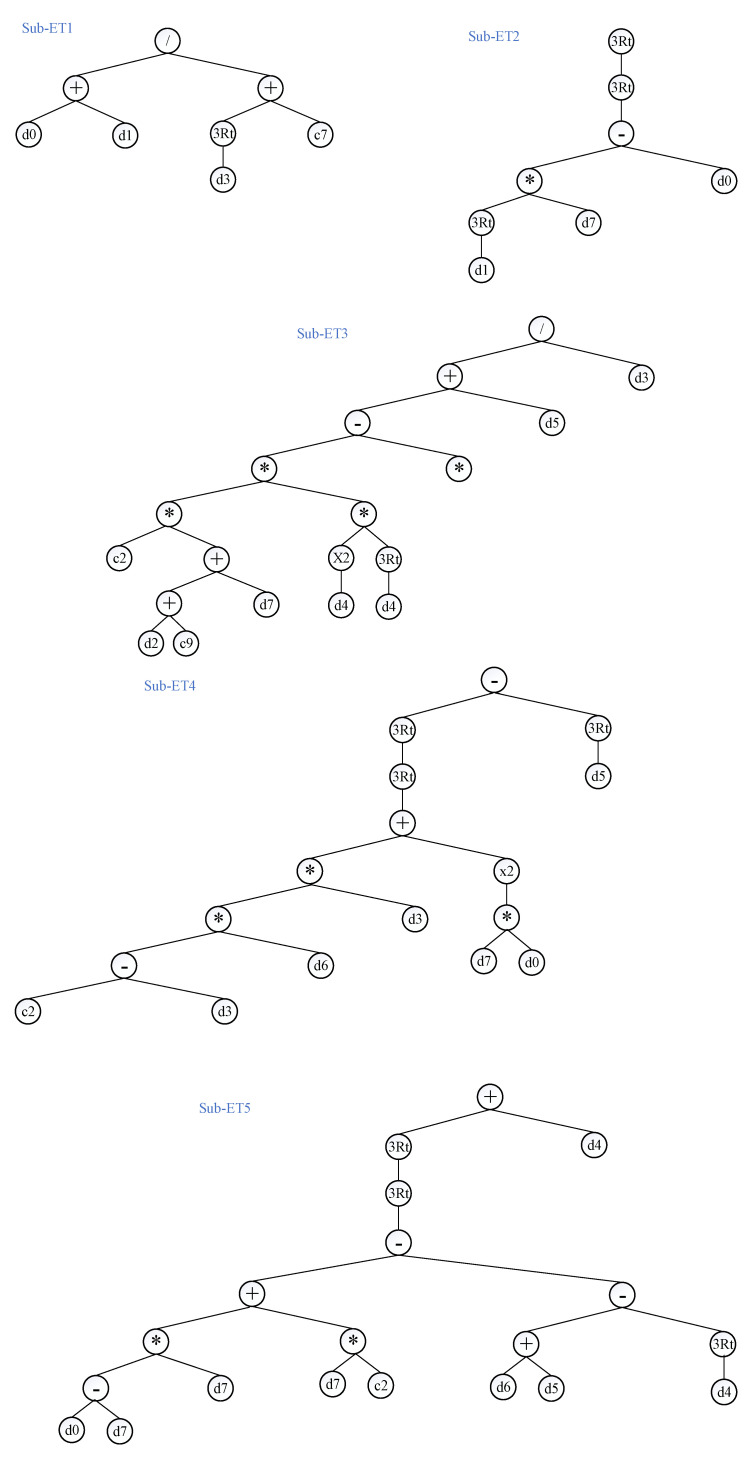
Expression trees derived from the best GEP model (* shows multiplication sign).

Figure 5 displays the performance of the models with changes in head size. A similar scenario was observed with an increasing number of chromosomes and increasing head size. An initial increase in the head size reduced R and corresponding increases in MAE, RMSE, and RSE for the training and validation values; however, a further increase in head size to 12 resulted in optimum results. Minimum values of errors and maximum values of correlation were observed for a head size of 12. While observing the performance of the models with the number of genes (Figure 6), it was observed that the maximum values of R were obtained at five genes as 0.90 and 0.871 for the training and validation data, respectively. The corresponding error indices were also observed to be a minimum for the five genes. A further increase in the number of genes may result in improved model performance; however, this was not investigated because it complicates the output mathematical equation. Hence, this study achieved optimum performance at 200 chromosomes, 12 head sizes, and five genes, as reflected in Table 5 for the GEP model. Mousavi et al. [34] also found 200 chromosomes as optimum hyperparameters along with three genes and eight head sizes while investigating the compressive strength of high-performance concrete. Therefore, this optimized model was used to extract the expression trees (ETs) (Figure 7), which were later used to develop the empirical equation presented in Section 3.1.2.

As previously mentioned, the GEP algorithm was allowed to randomly partition the training and validation data. By doing so, the models generated during the training process tend to overfit, enhancing the performance of the training set while decreasing the validation data performance [44]. To avoid this issue, Gandomi et al. [45] suggested selecting a model with the minimum objective function (OF) expressed as Equation (7). The magnitude of the OF ranges from 0 to the maximum, whereas a value approaching zero implies a relatively better model [46]. The value of R ranges between 0 and 1, where 1 reflects a perfect correlation, whereas a value of zero shows no correlation between the inputs and the target variable. It has also been reported that *R* exceeding 0.8 represents a more robust prediction of the forecasted values [47]

(1) $$R=\frac{\sum_{i=1}^{n}\left(e_{i}-{\bar{e}}_{i\;}\right)\left(m_{i}-{\bar{m}}_{i\;}\right)}{\sqrt{\sum_{i=1}^{n}{\left(e_{i}-{\bar{e}}_{i\;}\right)}^{2}{\left(m_{i}-{\bar{m}}_{i\;}\right)}^{2}}}$$

(2) $$MAE=\frac{\sum_{i=1}^{n}|e_{i}-m_{i}|}{n}$$

(3) $$RMSE=\sqrt{\frac{\sum_{i=1}^{n}{\left(e_{i}-m_{i\;}\right)}^{2}}{n}}$$

(4) $$RSE=\frac{\sum_{i=1}^{n}{\left(e_{i}-m_{i\;}\right)}^{2}}{\sum_{i=1}^{n}{\left(\bar{e}-m_{i\;}\right)}^{2}}$$

(5) $$RRMSE=\frac{1}{\left|\bar{e}\right|}\sqrt{\frac{\sum_{i=1}^{n}{\left(e_{i}-m_{i\;}\right)}^{2}}{n}}$$

(6) $$\rho =\frac{RRMSE}{\left(1+R\right)}$$

(7) $$OF=\left(\frac{n_{T}-n_{v}}{n}\right)\rho_{T}+2\left(\frac{n_{v}}{n}\right)\rho_{V}$$

where *e_i_* and *m_i_* are the nth experimental and model CS (%), respectively; $\bar{e_{i\;}}$ and $\bar{m_{i}}$ denote the average values of the experimental and model CS (%), respectively; and *n* is the number of samples in the dataset. The subscripts *T* and *V* in Equation (7) represent the training and validation data, respectively, and *n* is the total number of sample points.

#### 2.2.3. Gradient Boosting Tree (GBT) Model

**Figure 8:**
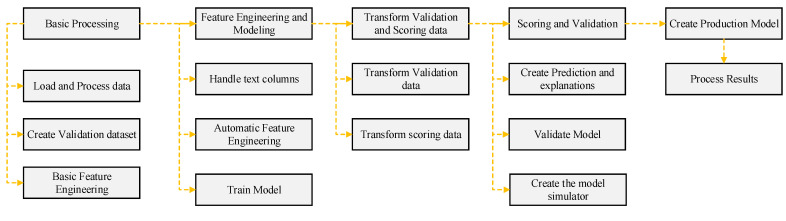
Flow diagram depicting GBT modeling.

The GBT model was developed in the RapidMiner environment by employing several basic steps of model development at the RapidMiner interface. Figure 8 shows the modeling process initiating from basic processing, along with other necessary steps, namely, transform validation and scoring data, feature modeling, and validation of the models. Moreover, scores, weights, and simulators were generated; the model was produced and the results delivered.

The hyperparameters of the GBT model were then optimized. The initial values of the GBT hyperparameters—maximal depth, number of trees, and learning rate—randomly began with lower bounds of 2, 30, and 0.001, respectively. The optimum performance of the developed model was achieved for 150 trees, 7 maximal depths, and a learning rate of 0.1, as shown in Table 6.

## 3. Results and Discussions

### 3.1. Predictive Performance and Validation

#### 3.1.1. Performance of Adaptive Neurofuzzy Inference System Model

**Figure 9:**
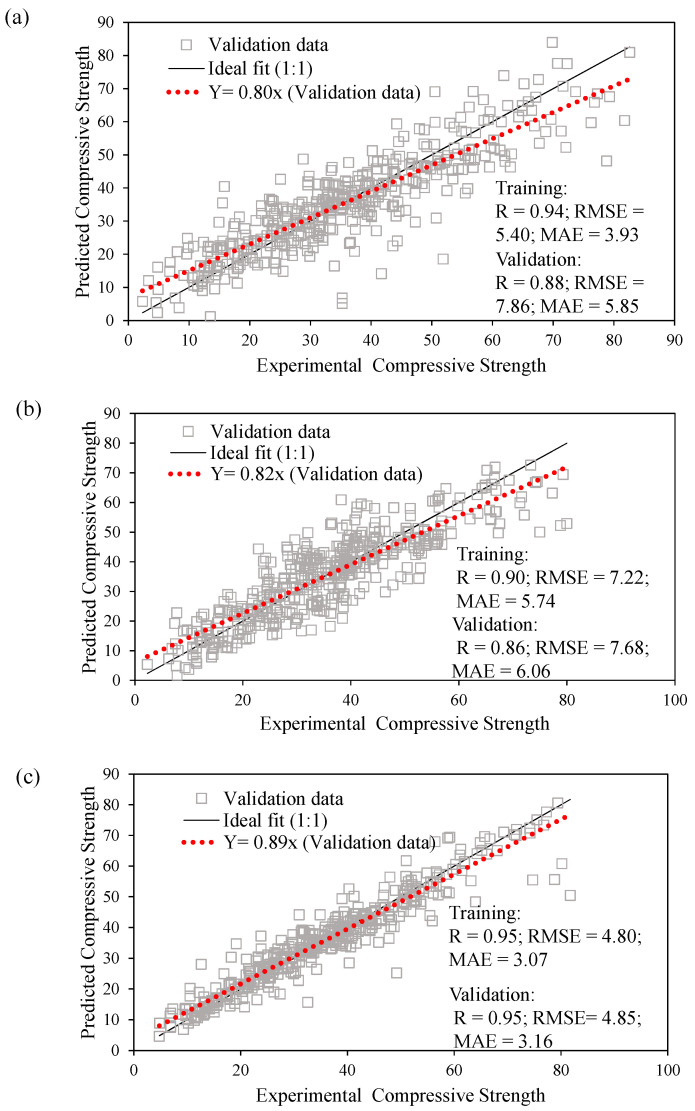
Comparison of experimental and predicted results: (**a**) ANFIS, (**b**) GEP, (**c**) GBT.

**Figure 10:**
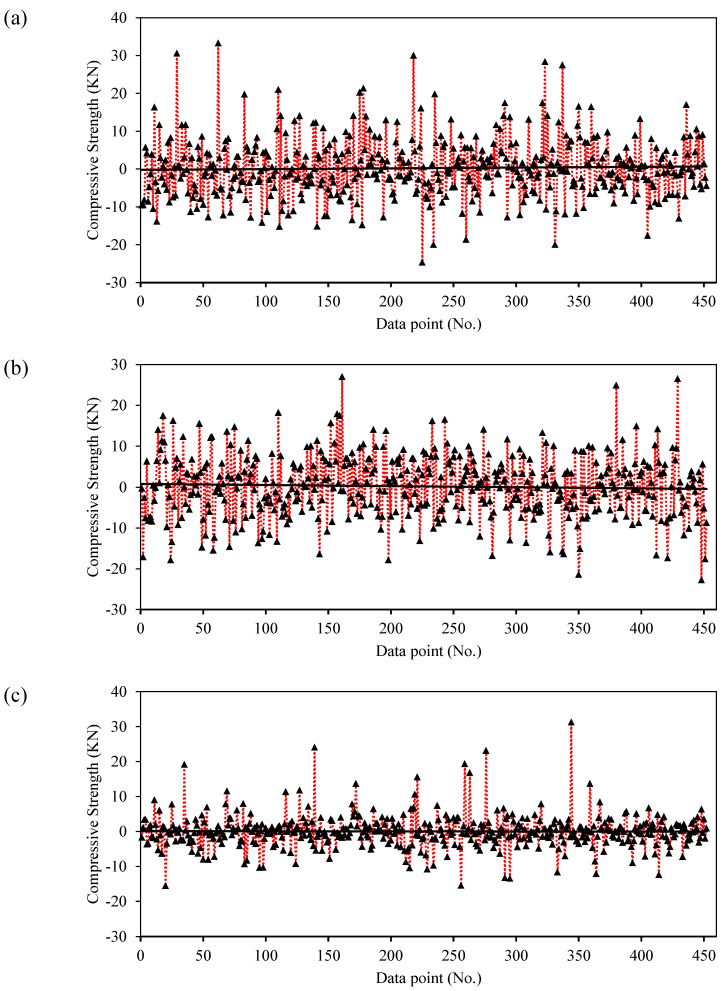
Error analysis: (**a**) ANFIS, (**b**) GEP, and (**c**) GBT.

**Figure 11:**
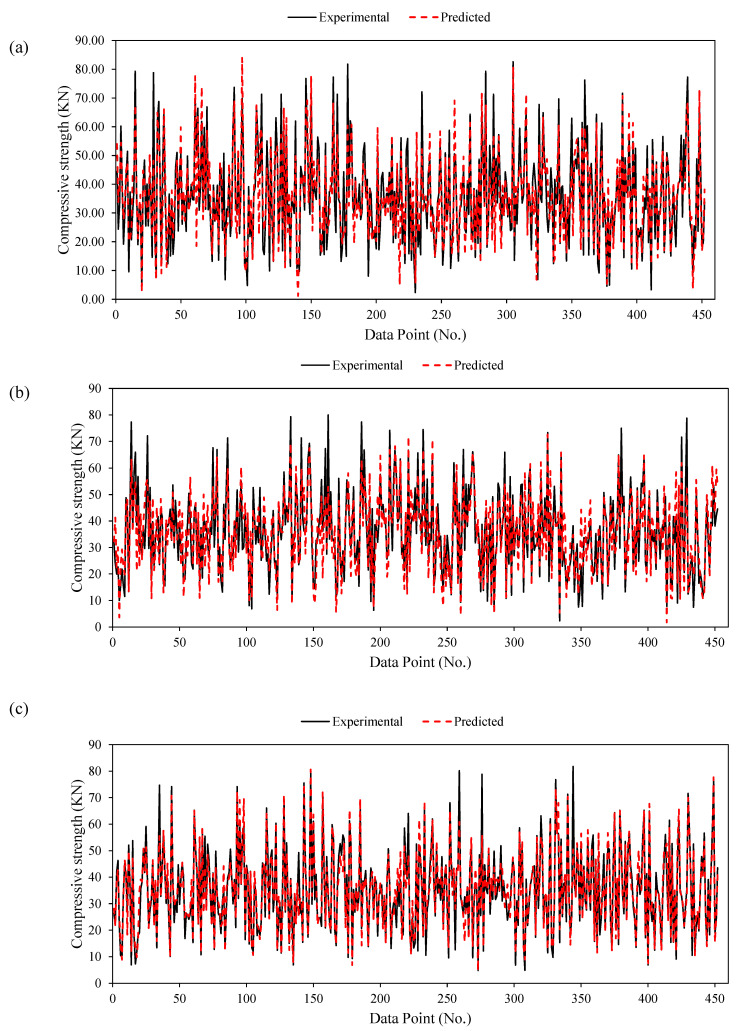
Tracing of experimental results by the predictions: (**a**) ANFIS, (**b**) GEP, and (**c**) GBT.

Figure 9a depicts a graphical representation of the experimental and ANFIS-modeled compression strength values. The closer the points are to the regression line (1:1 plot), the more efficacious an AI model is [48]. According to Madandoust et al. [49], while assessing the in situ strength of concrete, the ANFIS technique has a significant ability to forecast its compressive strength. However, ANFIS yields higher accuracy than conventional ANN models, and they exhibit the problem of overfitting [50]. Furthermore, the ANFIS model considered in this study precisely captures the effect of all input factors for the estimation of the compressive strength of concrete. In the case of training and validation datasets, the coefficients of correlation are 0.94 and 0.88, respectively, thus indicating a strong correlation between the experimental and ANFIS-modeled values [46]. However, the value of R is insensitive to both the multiplication and division of the resulting compressive strength [51]; hence, the values of the RMSE and MAE parameters were determined to assess the performance of the final ANFIS model. This is also evident from the lower values of the performance indices (i.e., RMSE_training_ = 5.40, RMSE_validation_ = 7.86, MAE_training_ = 3.93, MAE_validation_ = 5.85). This suggests that the concentration of the error scatter is mainly near zero. In addition, the error analysis graph in Figure 10a shows that the compressive strength errors range from −25 to 33 MPa, which mainly occurred in the dataset points of the training set. On the contrary, the validation dataset of ANFIS model can be seen to have a smaller range of errors. Armaghani et al. [50] explained the issue of overfitting in the ANFIS models. Finally, from Figure 11a, it can be observed that the tracing of the experimental versus ANFIS-modeled values exhibits significantly greater nonconformity, particularly in two regions, that is, data points ranging from 60 to 90 and 200 to 300.

#### 3.1.2. Performance of Gene Expression Programming Model

According to Ferreira [52], gene language and language of expression trees (ETs) are deeply interrelated. ETs present a variety of problems in the genetic programming approach, such as functions, constants, operators, and different variables [53]. To determine a simple mathematical formula to compute the compressive strength, typical ETs for the GEP algorithm of the formulated models were developed, as shown in Figure 7. The sub-ETs of the compressive strength of concrete incorporated six fundamental mathematical functions: +, −, x, ÷, 3Rt, and x^2^. The ETs were decoded after the GEP model was developed to derive a simple mathematical formula for the compressive strength in terms of input variables. According to the hyperparameter settings of the GEP model, the Karva notation or K-expression [45] was deployed to change the ETs into simple mathematical expressions (as shown in Equations (8)–(13)):

(8) $$f_{c}^{\prime }=A+B+C+D+E$$

(9) $$A=\frac{\left(C+GGBFS\right)}{\sqrt[{3}]{W}+7.07}$$

(10) $$B=\sqrt[{3}]{\sqrt[{3}]{-C+A\cdot \sqrt[{3}]{GGBFS}}}$$

(11) $$C=\left(\frac{\left(8.12\left(\left(FA+8.59\right)+A\right)\right)-\left(\left(SP^{2}\right)\cdot \sqrt[{3}]{SP}\right))+CA}{W}\right)$$

(12) $$D=\sqrt[{3}]{\sqrt[{3}]{\left(\left(-7.43-W\right)FAgg\right)W+{\left(A\cdot C\right)}^{2}-\sqrt[{3}]{CA}}}$$

(13) $$E=\sqrt[{3}]{\sqrt[{3}]{\left((C-A\right)A)-6.27A-\left(FAgg+CA\right)-\sqrt[{3}]{A}}+SP}$$

where *C* = cement; *GGBFS* = ground granulated blast furnace slag; *FA* = fly ash; *W* = water; *SP* = superplasticizer; *CA* = coarse aggregate; *FAgg* = fine aggregate; *A* = age.

These equations can be employed to forecast the compressive strength of concrete [54].

Figure 9b illustrates the experimental and forecasted compressive strength values using the proposed GEP model. Note that, similar to the case of ANFIS modeling, the results of the training dataset (R = 0.90) are superior to those of the validation datasets (R = 0.86), implying that the results of modeling with ANFIS are comparatively better. At higher R values, a strong correlation can be observed between the input parameters [55]. Furthermore, the RMSE and MAE performance indices show small differences in the training and validation datasets, that is, (7.22 and 7.68) and (5.74 and 6.06), respectively. The error analysis (Figure 10b) shows that the errors in the compressive strength by GEP modeling ranged from −23 to 28 MPa. Finally, the tracing of experimental and GEP-modeled compressive strength values in Figure 11b reveals a greater deviation over the entire dataset, which suggests the unsuitability of GEP for this particular dataset, that is, ANFIS > GEP.

#### 3.1.3. Performance of Gradient Boosting Tree Model

Figure 9c shows a visual comparison of the experimental and estimated compressive strength values using the developed GBT forecasting model, which outperforms the ANFIS and GEP models across all three performance indices (R, RMSE, and MAE) considered in the current study. The training and validation datasets possessed similar R values of 0.95. However, a slight difference is observed in the MAE indices (3.07 and 3.16) as compared with the RMSE values, i.e., 4.80 and 4.85, for the training and validation sets, respectively. In addition, the percentage decreases in RMSE values of GB-based modeling training and validation datasets in comparison with ANFIS and GEP modeling is (11% and 33.02%) and (62% and 58%), respectively. In contrast, the MAE decreased by (21.88% and 46.52%) and (46% and 48%), respectively. Correspondingly, the error analysis graph (Figure 10c) shows that the compressive strength has errors in the range −18 to 31 MPa. Finally, the tracing of experimental and GBT-modeled values (Figure 11c) illustrates that, unlike the ANFIS and GEP models, the variation in the values is less, thereby yielding the best results, that is, GB > ANFIS > GEP.

### 3.2. Comparison of the Models

**Figure 12:**
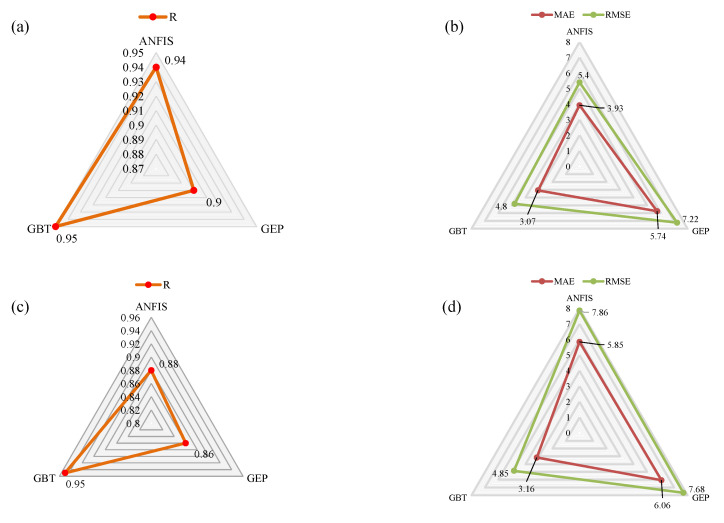
Comparison of the developed models based on various statistical evaluation indices using radar plots. R values (**a**,**c**); MAE and RMSE values (**b**,**d**).

A comparison of the models is presented in the form of a radar plot shown in Figure 12. The statistical evaluation of models in the case of multidimensional problems is a complex scenario; however, radar plots simplify the analysis. A radar plot typically consists of a group of variables expressed as vertices of the radar plot, and the count of the performance index is shown on a scale along the spoke. The spokes in the plot are aligned such that they resemble a clock. The shape of the chart resembles a triangle, quadrilateral, or pentagon, depending on the number of variables in the group. Figure 12a,c depicts the comparison of the three models in the form of the correlation coefficient (R) for the training and validation data. For the training data, the GBT model excels in the case of R values yielding a magnitude of 0.95 for the training and 0.95 for the validation data set. The ANFIS and GEP models yielded R values (0.94 and 0.88) and (0.90 and 0.86), for training and validation, respectively. The observed values of correlation for all models depict strong correlations between the input attributes and the output variable; however, the GBT model shows outclass performance. The value of R in the validation data for the GBT model was precisely equal to that of the training data, suggesting no overfitting of the model during the training process. In contrast, the values of R for the ANFIS and GEP models were slightly lower than those of the training data, depicting marginally over the fitness of the developed ANFIS and GEP models.

While investigating Figure 12b,d, it can be observed that the MAE values are smaller than the RMSE values, corroborating the statistical evidence [56]. Similar to the correlation comparison, the value of MAE was observed as 3.07 and 3.16 for the training and validation data of the GBT model. The magnitudes of MAE for the ANFIS and GEP models were observed to be (3.93, 5.85) MPa and (5.74, 6.06) MPa for the training and validation data, respectively. A similar trend was observed for the RMSE comparison; minimum values were observed for the GBT model equaling 4.80 and 4.85 MPa for the training and validation sets, respectively. The error analysis shows that the model interpreted an acceptable level of error (<20%) for almost the majority of data points; however, the GBT models interpreted the minimal error observed from the MAE and RMSE analyses. Owing to the superior performance of the model, the sensitivity and parameters were based on the GBT model discussed in the following section.

#### Comparison with the Literature

The developed GBT model was compared in detail with models available in the literature (Table 7). The same statistical indices were used to evaluate the models in the literature. Farooq et al. [57] conducted a comprehensive study on the estimation of compressive strength using ensemble models, such as DT and MPNN, optimized by AdaBoost, Bagging, and XgBoost. The most accurate model manifested an MAE of 3.71 MPa and an RMSE of 5.17 MPa, which in the current study were 3.07 and 4.80 MPa, respectively. Table 7 further shows that the results from the GBT model excel in all the developed models presented in this study.

### 3.3. Parametric and Sensitivity Analysis

**Figure 13:**
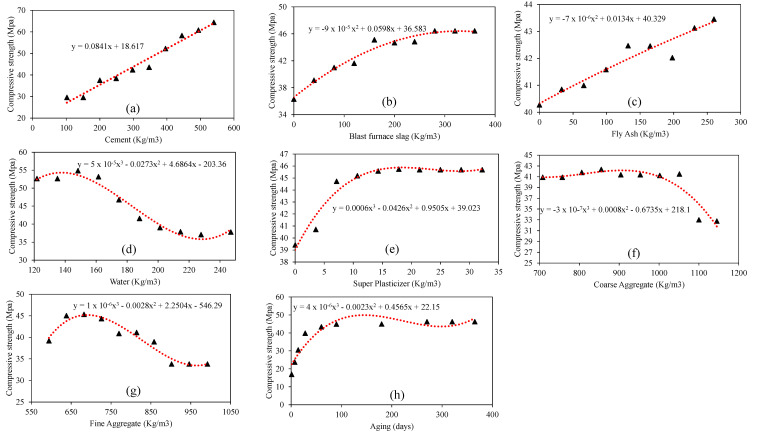
Parametric analysis of the GBT model i.e. variation of compressive strength with. (**a**) Cement (**b**) Blast furnace slag (**c**) Fly Ash (**d**) water (**e**) superplasticizer (**f**) Coarse Aggregates (**g**) Fina Aggregates (**h**) Age of testing.

A parametric analysis was conducted as a second level validation of the developed GBT model, alongside the investigation of the variables contributing to the compressive strength of green concrete. Following the descriptive statistics of the employed database, a variable was varied between its extreme values while keeping other variables constant at their average magnitudes to generate the simulated dataset listed in Table 8. In this way, its contribution toward compressive strength was captured with the varying input attributes illustrated in Figure 13.

In Figure 13a, it can be observed that with increasing cement content (100–550 kg/m^3^), the compressive strength of concrete increased linearly up to 65 MPa. Mohammed et al. [59] reported similar observations regarding the enhancement of the mechanical properties of concrete upon blending with cement particles. As shown in Figure 13b, the GGBFS significantly improves the compressive strength such that the inclusion of 250 kg/m^3^ of slag yields maximum compression strength of approximately 46 MPa, and upon further increase in the slag (up to 350 kg/m^3^), the strength value remains constant. According to Lubeck et al. [60], compressive strength values of 35–60 MPa were recorded for a blend of 50 percent blast furnace slag and 50 percent cement. The results of the compressive strength versus fly ash dosage in Figure 13c show a linearly increasing trend, which is in accordance with the findings of [61]. In Figure 13d,g, the impacts of increasing water and fine aggregates on the compressive strength are identical, wherein the highest strength values (55 and 45 MPa, respectively) can be observed in the initial stages (140 and 680 kg/m^3^, respectively), whereas these values plummet upon further increases in water and fine aggregates. Furthermore, both cases exhibited constant compressive strength values in the later stages (>220 and >950 kg/m^3^, respectively). Donza et al. [62] argued that the crushed sand particle form and structure significantly impact the bonding of the paste and aggregate fragments, resulting in increased concrete strength. In Figure 13e,h, the impact of increasing the superplasticizer and aging on the compressive strength exhibits almost similar trends, wherein the highest strength values (46 and 50 MPa, respectively) can be observed in the initial stages (15 kg/m^3^ and 110 days, respectively), while these values are constant with further increases in the superplasticizer and aging. Compressive strength models have been suggested to be a function of age at different curing periods [63]. In addition, Beshr et al. [64] stated that the compressive strength of a variety of aggregates increased to 60 MPa after of 180 days curing. Further research is needed to study the behavior of compression strength with aging, particularly after 260 days and more. Finally, Figure 13f shows that the increase in compressive strength is almost constant when the amount of coarse aggregates is up to 1000 kg/m^3^; however, the strength values plummet upon further addition of coarse aggregates. The type of aggregate used to yield high-strength concrete affects the strength, stiffness, and fracture energy of concrete for a specific water/cement ratio (W/C) [65]. According to Beshr et al. [64], the modulus of elasticity of concrete is also influenced by the quality of the coarse aggregate such that weaker aggregates form ductile concrete more readily than stronger aggregates.

**Figure 14:**
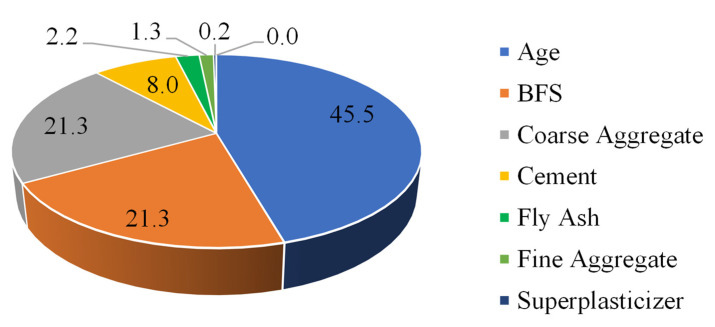
Relative contribution (%) of input variables in yielding compressive strength of green concrete.

The sensitivity analysis (Figure 14) showed that the aging of concrete is the most influential parameter contributing to the compressive strength of green concrete, followed by the addition of GGBFS, coarse aggregates, and cement, and others.

## 4. Conclusions

Using FA and GGBFS to produce green concrete, this study presents the most accurate prediction model using the GBT algorithm to estimate the compressive strength. The performance of the developed GBT model was extensively compared with other AI techniques, namely, the GEP and ANFIS models. Moreover, the performance of the GBT model was compared with that of existing AI models in the literature. The following conclusions were drawn from this study.

1. During model training, it was determined that the optimum results of the ANFIS models were achieved by designing a subclustering hybrid FIS with an aspect ratio of 0.5. While assessing the effect of various genetic parameters on the performance of the developed GEP models, it was initially evaluated that an increase in the number of chromosomes, genes, and head size from 30–50 to 3–4 and 8–10 reduced the performance; however, further increases yielded optimum results, and maximum correlation and maximum error indices were achieved for 200 chromosomes, 5 genes, and head size 10.
2. For GBT modeling, the learning rate, number of trees, and maximal depth were varied from their lower bounds of 0.001, 30, and 2, respectively. These setting parameters were slowly changed until the best results were obtained for learning rate 0.1, 150 trees, and maximal depth 7.
3. All three models exhibited a strong agreement between the input attributes and the output variable: ANFIS yielded R = 0.94, MAE = 3.93 MPa, and RMSE = 5.4 MPa; GEP resulted R= 0.90, MAE = 5.74, and RMSE = 7.22 MPa; and the GBT model gave the best performance in the form of the highest correlation (R = 0.95, MAE = 3.07, and RMSE = 4.80). This reflects the order of accuracy of the developed models: GBT > ANFIS > GEP. The GBT model was also compared with the existing models in the literature, suggesting that GBT is a more accurate model.
4. The parametric study showed that the compressive strength increased linearly with the increase in the amount of cement at a constant amount of water equaling 182.98 kg/m^3^. An increase in the amount of GGBFS beyond 250 kg/m^3^ had no significant effect on the compressive strength at a constant input cement quantity of 276 kg/m^3^, fly ash of 62.81 kg/m^3^, and 182 kg/m^3^ of water. This suggests that the optimum ratio of GGBFS as 0.42 to binder content at a water-to-binder ratio of 0.31. The study also concluded that increasing the superplasticizer beyond 2.9% of the binder content at a water-to-binder ratio of 0.44 has no significant impact on the compressive strength of concrete.
5. The gain of compressive strength with the variation in aging of concrete depicts a much steeper slope of strength at the beginning, which almost becomes flat after 100 days. This observation confirms the validation of the developed GBT model, as the strength of concrete varies rapidly at the beginning. Moreover, the sensitivity analysis depicted the age of concrete as the most influential parameter contributing to compressive strength, followed by the addition of blast furnace slag and the quantity of coarse aggregates.

AI models developed in this study were based on an experimental database taken from the existing literature. New experiments shall be conducted for investigating the validity of the trained models on the entire new dataset. Moreover, new hybrid models shall be developed for investigating the compressive strength of green concrete on the basis of new experimental data.

## Figures and Tables

**Table 1:** Statistical functions for input and output parameters.

Parameter	Cement	Ground Granulated Blast Furnace Slag	Fine Aggregates	Water	Superplasticizer	Coarse Aggregates	Fly Ash	Age	Concrete Compressive Strength
Symbol	C	GGBFS	FAgg	W	SP	CA	FA	Age	$f_{c}^{\prime }$
Unit	(Kg/m^3^)							(Days)	(MPa)
Minimum	102	0	0	121.75	0	708	594	1	2.33
Maximum	540	359.4	260	247	32.2	1145	992.6	365	82.60
Mean	276.50	74.27	62.81	182.98	6.42	964.83	770.49	44.06	35.84
Median	266	26	0	185.7	6.7	966.8	777.5	28	34.6737
SD	103.47	84.25	71.58	21.71	5.80	82.79	79.37	60.44	16.10
Kurtosis	−0.4598	−0.4845	−0.9091	0.0736	1.4571	−0.3953	−0.1659	13.8117	−0.1564
Skewness	0.5292	0.7689	0.6058	0.0888	0.8361	−0.1674	−0.1890	3.4696	0.4224

**Table 2:** Correlation matrix.

	C	BFS	FAgg	W	SP	CA	FA	Age	$f_{c}^{\prime }$
C	1								
BFS	−0.27275	1							
FA	−0.42043	−0.28889	1						
W	−0.08895	0.09949	−0.15086	1					
SP	0.06772	0.05283	0.35272	−0.58810	1				
CA	−0.07299	−0.26806	−0.10552	−0.27084	−0.27498	1			
FAgg	−0.18588	−0.27598	−0.00626	−0.42471	0.19830	−0.15341	1		
Age	0.09061	−0.04422	−0.16314	0.24202	−0.19843	0.02328	−0.13945	1	
$f_{c}^{\prime }$	0.48859	0.11985	−0.06440	−0.27821	0.35551	−0.15485	−0.16523	0.32386	1

Note: All input parameters are measured in kg/m^3^, except age of testing, which is measured in days. The compressive strength of concrete is in MPa.

**Table 3:** Setting hyperparameters for the ANFIS model.

Parameter	Setting
Sampling	
Training record	681
Validation/testing	452
General	
Type	Sugeno
Number of nodes	353
Number of linear parameters	171
Number of nonlinear parameters	304
Number of fuzzy rules	19
And Method	prod
Imp Method	prod
Or Method	probor
Agg Method	Sum
Defuzzification Method	whatever
FIS properties	
FIS type	Sub clustering
Training FIS method	hybrid
Range of influence	0.5
Squash factor	1.25
Aspect ratio	0.5
Error tolerance	0
Epochs	100

**Table 4:** Setting parameters for the GEP models.

Parameter	Setting
Sampling	
Training record	681
Validation/testing	452
General	
Genes	3, 4, 5
Number of chromosomes	30, 50, 100, 200
Head size	8, 10, 12
Linking function	Addition
Function set	+, −, *, /, x^(1/3)^, x^2^
Numerical constants	
Constants per gene	10
Data type	Floating number
Upper bound	10
Lower bound	−10
Genetic operators	
Mutation rate	0.00138
Fixed root mutation rate	0.00068
Function insertion rate	0.00206
Inversion rate	0.00546
IS transposition rate	0.00546
RIS transposition rate	0.00546
Gene composition rate	0.00277
Gene transposition rate	0.00277

**Table 5:** Details of trials undertaken for hyperparameter selection for the GEP model.

Variable Setting Parameters			Training Data Set				Validation Data Set			
Model No.	Fitness Function	Number of Chromosomes, Head Size, Genes	Correlation (R)	RMSE	MAE	RSE	Correlation (R)	RMSE	MAE	RSE
GEP1	RMSE	30, 8, 3	0.876	7.96	6.25	0.233	0.855	8.12	6.17	0.276
GEP2	RMSE	50, 10, 4	0.857	8.51	6.54	0.266	0.817	9.03	7.01	0.341
GEP3	RMSE	100, 10, 5	0.878	7.92	6.10	0.231	0.855	8.24	6.32	0.284
GEP4	RMSE	200, 12, 5	0.90	7.22	5.74	0.191	0.871	7.67	6.06	0.246

**Table 6:** Optimization of the GBT model.

Model	Parameter	Value	Error Rate Optimization (%)
GBT	Number of trees, maximum depth, learning rate	30, 2, 0.001	28.90
		90, 2, 0.001	28.36
		150, 2, 0.001	27.87
		30, 4, 0.001	28.80
		90, 4, 0.001	28.10
		150, 4, 0.001	27.41
		30, 7, 0.001	28.73
		90, 7, 0.001	27.88
		150, 7, 0.001	27.11
		30, 2, 0.01	26.83
		90, 2, 0.01	23.60
		150, 2, 0.01	21.32
		30, 4, 0.01	25.82
		90, 4, 0.01	21.27
		150, 4, 0.01	18.43
		30, 7, 0.01	25.33
		90, 7, 0.01	20.34
		150, 7, 0.01	17.27
		30, 2, 0.1	17.72
		90, 2, 0.1	13.96
		150, 2, 0.1	13.21
		30, 4, 0.1	14.86
		90, 4, 0.1	12.49
		150, 4, 0.1	12.12
		30, 7, 0.1	13.38
		90, 7, 0.1	11.82
		**150, 7, 0.1**	11.63

**Table 7:** Comparison of the performance of the developed models with those previously reported in the literature.

Model	Abbreviation	RMSE (MPa)	MAE (MPa)	R	References
Decision tree	DT	7.37	4.62	0.81	[57]
Multilayer perceptron neuron network	MPNN	6.67	5.14	0.8	
Support vector regression	SVR	7.17	5.56	0.81	
Decision tree—Adaboost	DT-Ab	5.22	3.69	0.91	
Multilayer perceptron neuron network—Adaboost	MPNN-Ab	6.25	4.6	0.85	
Support vector regression—Adaboost	SVR-Ab	7.01	5.07	0.82	
Random forest	RF	4.6	3.23	0.92	
Decision tree—Bagging	DT-B	4.72	3.37	0.92	
Multilayer perceptron neuron network—Bagging	MPNN-B	6.66	4.88	0.84	
Support vector regression—Bagging	SVR-B	7.01	5.15	0.84	
Decision tree—Xgboost	DT-Xgb	5.17	3.71	0.9	
Multilayer perceptron neuron network—Xgboost	MPNN-Xgb	517	3.71	0.88	
Support vector regression—Xgboost	SVR-Xgb	5.17	3.71	0.9	
Gradient boosting tree	**GBT^+^**	**4.8**	**3.07**	**0.95**	Present study
Gene expression programming	GEP^+^	7.22	5.74	0.9	
Adaptive neurofuzzy inference system	ANFIS^+^	5.4	3.93	0.94	
Gene expression programming	GEP		5.2	0.9	[34]
Artificial neural network	ANN	6.329	4.421	0.93	[29]
Ensemble model artificial neural network—supportvector regression	ANN-SVR	6.17	4.24	0.94	
Chi-squared automatic interaction detector	CHAID	8.98	6.088	0.86	
Linear regression	LR	11.24	7.87	0.80	
Generalized linear model	GENLIN	11.37	7.87	0.80	
Classification and regression trees	CART	9.703	6.815	0.84	[29]
Smart firefly algorithm-based least squares	SFA-LSSVR	5.62	3.86	0.94	[58]
Modified firefly algorithm-based ANN	MFA-ANN	5.82	3.41	0.93	[26]

Note: ^+^ signs show the models developed in present study.

**Table 8:** Simulated dataset for parametric and sensitivity analysis.

Variable Input Parameters		No. of Data Points	Constant Input Parameters
Parameter	Range		
C	102–540	10	GGBFS = 74.27, FA = 62.81, W = 182.98, SP = 6.42, CA = 964.83, FAgg = 770.49, A = 44.06
GGBFS	0–359.40	10	C = 276, FA = 62.81, W = 182.98, SP = 6.42, CA = 964.83, FAgg = 770.49, A = 44.06
FA	0–260	10	C = 276, GGBFS = 74.27, W = 182.98, SP = 6.42, CA = 964.83, FAgg = 770.49, A = 44.06
W	121.75–247	10	C = 276, GGBFS = 74.27, FA = 62.81, SP = 6.42, CA = 964.83, FAgg = 770.49, A = 44.06
SP	0–32.20	10	C = 276, GGBFS = 74.27, FA = 62.81, W = 182.98, CA = 964.83, FAgg = 770.49, A = 44.06
CA	708–1145	10	C = 276, GGBFS = 74.27, FA = 62.81, W = 182.98, SP = 6.42, FAgg = 770.49, A = 44.06
FAgg	594–992	10	C = 276, GGBFS = 74.27, FA = 62.81, W = 182.98, SP = 6.42, CA = 964.83, A = 44.06
A	1–365	10	C = 276, GGBFS = 74.27, FA = 62.81, W = 182.98, SP = 6.42, CA = 964.83, FAgg = 770.49

## Data Availability

All the data used in the manuscript has been cited properly.

## References

[1] Yang K.-H., Jung Y.-B., Cho M.-S., Tae S.-H. (2015). Effect of supplementary cementitious materials on reduction of CO_2_ emissions from concrete. J. Clean. Prod..

[2] Holger W., Ostermeyer Y., Salzer C., Escamilla E.Z. (2012). Indicator based sustainability assessment tool for affordable housing construction technologies. Ecol. Indic..

[3] Barcelo L., Kline J., Walenta G., Gartner E. (2014). Cement and carbon emissions. Mater. Struct..

[4] Miller S.A., John V.M., Pacca S.A., Horvath A. (2018). Carbon dioxide reduction potential in the global cement industry by 2050. Cem. Concr. Res..

[5] Zhongming Z., Linong L., Xiaona Y., Wangqiang Z., Wei L. (2018). Cement technology roadmap shows how the path to achieve CO_2_ reductions up to 24% by 2050. World Business Council for Sustainable Development.

[6] CSI–Cement Sustainability Initiative. Global Cement Database on CO_2_ and Energy Information.

[7] Ribeiro F.R.C., Modolo R.C.E., Kulakowski M.P., Brehm F.A., Moraes C.A.M., Ferreira V.M., Mesquita E.F.T., de Azevedo A.R.G., Monteiro S.N. (2022). Production of belite based clinker from ornamental stone processing sludge and calcium carbonate sludge with lower CO_2_ emissions. Materials.

[8] Ahmed Ali K., Ahmad M.I., Yusup Y. (2020). Issues, impacts, and mitigations of carbon dioxide emissions in the building sector. Sustainability.

[9] Winters D., Boakye K., Simske S. (2022). Toward carbon-neutral concrete through biochar–cement–calcium carbonate composites: A critical review. Sustainability.

[10] Poudyal L., Adhikari K. (2021). Environmental sustainability in cement industry: An integrated approach for green and economical cement production. Resour. Environ. Sustain..

[11] Salami B.A., Maslehuddin M., Mohammed I. (2015). Mechanical properties and durability characteristics of scc incorporating crushed limestone powder. J. Sustain. Cem. Based Mater..

[12] Ankur N., Singh N. (2021). Performance of cement mortars and concretes containing coal bottom ash: A comprehensive review. Renew. Sustain. Energy Rev..

[13] Gençel O., Karadag O., Oren O.H., Bilir T. (2021). Steel slag and its applications in cement and concrete technology: A review. Constr. Build. Mater..

[14] Mehta A., Ashish D.K. (2020). Silica fume and waste glass in cement concrete production: A review. J. Build. Eng..

[15] Lemougna P.N., Wang K.-T., Tang Q., Nzeukou A., Billong N., Melo U.C., Cui X.-M. (2018). Review on the use of volcanic ashes for engineering applications. Resour. Conserv. Recycl..

[16] Scrivener K., Martirena F., Bishnoi S., Maity S. (2018). Calcined clay limestone cements (LC^3^). Cem. Concr. Res..

[17] Tayeh B.A., Alyousef R., Alabduljabbar H., Alaskar A. (2021). Recycling of rice husk waste for a sustainable concrete: A critical review. J. Clean. Prod..

[18] Raheem A.A., Ikotun B.D. (2020). Incorporation of agricultural residues as partial substitution for cement in concrete and mortar—A review. J. Build. Eng..

[19] Jiang Y., Ling T.-C., Mo K.H., Shi C. (2019). A critical review of waste glass powder—Multiple roles of utilization in cement-based materials and construction products. J. Environ. Manag..

[20] Norhasri M.M., Hamidah M., Fadzil A.M. (2017). Applications of using nano material in concrete: A review. Constr. Build. Mater..

[21] Giergiczny Z. (2019). Fly ash and slag. Cem. Concr. Res..

[22] Akpinar P., Khashman A. (2017). Intelligent classification system for concrete compressive strength. Procedia Comput. Sci..

[23] Jamal A., Al-Ahmadi H.M., Butt F.M., Iqbal M., Almoshaogeh M., Ali S. (2021). Metaheuristics for Traffic Control and Optimization: Current Challenges and Prospects.

[24] Sadowski Ł., Piechówka-Mielnik M., Widziszowski T., Gardynik A., Mackiewicz S. (2019). Hybrid ultrasonic-neural prediction of the compressive strength of environmentally friendly concrete screeds with high volume of waste quartz mineral dust. J. Clean. Prod..

[25] Singh P., Bhardwaj S., Dixit S., Shaw R.N., Ghosh A., Mekhilef S., Favorskaya M., Pandey R.K., Shaw R.N. (2021). Development of prediction models to determine compressive strength and workability of sustainable concrete with ann. Innovations in Electrical and Electronic Engineering.

[26] Bui D.-K., Nguyen T., Chou J.-S., Nguyen-Xuan H., Ngo T.D. (2018). A modified firefly algorithm-artificial neural network expert system for predicting compressive and tensile strength of high-performance concrete. Constr. Build. Mater..

[27] Zarandi M.F., Türksen I.B., Sobhani J., Ramezanianpour A.A. (2008). Fuzzy polynomial neural networks for approximation of the compressive strength of concrete. Appl. Soft Comput..

[28] Chou J.-S., Chiu C.-K., Farfoura M., Al-Taharwa I. (2011). Optimizing the prediction accuracy of concrete compressive strength based on a comparison of data-mining techniques. J. Comput. Civ. Eng..

[29] Chou J.-S., Pham A.-D. (2013). Enhanced artificial intelligence for ensemble approach to predicting high performance concrete compressive strength. Constr. Build. Mater..

[30] Deepa C., SathiyaKumari K., Sudha V.P. (2010). Prediction of the compressive strength of high performance concrete mix using tree based modeling. Int. J. Comput. Appl..

[31] Erdal H.I., Karakurt O., Namli E. (2013). High performance concrete compressive strength forecasting using ensemble models based on discrete wavelet transform. Eng. Appl. Artif. Intell..

[32] Yeh I.-C., Lien L.-C. (2009). Knowledge discovery of concrete material using genetic operation trees. Expert Syst. Appl..

[33] Kaloop M.R., Kumar D., Samui P., Hu J.W., Kim D. (2020). Compressive strength prediction of high-performance concrete using gradient tree boosting machine. Constr. Build. Mater..

[34] Mousavi S.M., Aminian P., Gandomi A.H., Alavi A.H., Bolandi H. (2012). A new predictive model for compressive strength of hpc using gene expression programming. Adv. Eng. Softw..

[35] Chen W., Panahi M., Tsangaratos P., Shahabi H., Ilia I., Panahi S., Li S., Jaafari A., Ahmad B.B. (2019). Applying population-based evolutionary algorithms and a neuro-fuzzy system for modeling landslide susceptibility. Catena.

[36] Yuan Z., Wang L.-N., Ji X. (2014). Prediction of concrete compressive strength: Research on hybrid models genetic based algorithms and anfis. Adv. Eng. Softw..

[37] Nguyen T., Kashani A., Ngo T., Bordas S. (2019). Deep neural network with high-order neuron for the prediction of foamed concrete strength. Comput. Aided Civil. Infrastruct. Eng..

[38] Jang J.-S. (1993). Anfis: Adaptive-network-based fuzzy inference system. IEEE Trans. Syst. Man Cybern..

[39] Takagi T., Sugeno M. (1985). Fuzzy identification of systems and its applications to modeling and control. IEEE Trans. Syst. Man Cybern..

[40] Ferreira C. (2001). Gene expression programming: A new adaptive algorithm for solving problems. arXiv.

[41] Mitchell M. (1998). An Introduction to Genetic Algorithms.

[42] Faradonbeh R.S., Hasanipanah M., Amnieh H.B., Armaghani D.J., Monjezi M. (2018). Development of gp and gep models to estimate an environmental issue induced by blasting operation. Environ. Monit. Assess..

[43] Sakino K., Nakahara H., Morino S., Nishiyama I. (2004). Behavior of centrally loaded concrete-filled steel-tube short columns. J. Struct. Eng..

[44] Gandomi A.H., Alavi A.H. (2012). A new multi-gene genetic programming approach to nonlinear system modeling. Part I: Materials and structural engineering problems. Neural Comput. Appl..

[45] Gandomi A.H., Roke D.A. (2015). Assessment of artificial neural network and genetic programming as predictive tools. Adv. Eng. Softw..

[46] Iqbal M., Zhang D., Jalal F.E., Javed M.F. (2021). Computational ai prediction models for residual tensile strength of gfrp bars aged in the alkaline concrete environment. Ocean. Eng..

[47] Jalal F.E., Xu Y., Li X., Jamhiri B., Iqbal M. (2021). Fractal approach in expansive clay-based materials with special focus on compacted gmz bentonite in nuclear waste disposal: A systematic review. Environ. Sci. Pollut. Res..

[48] Saadat M., Bayat M. (2019). Prediction of the unconfined compressive strength of stabilised soil by adaptive neuro fuzzy inference system (anfis) and non-linear regression (nlr). Geomech. Geoengin..

[49] Madandoust R., Bungey J.H., Ghavidel R. (2012). Prediction of the concrete compressive strength by means of core testing using gmdh-type neural network and anfis models. Comput. Mater. Sci..

[50] Armaghani D.J., Asteris P.G. (2021). A comparative study of ann and anfis models for the prediction of cement-based mortar materials compressive strength. Neural Comput. Appl..

[51] Iqbal M.F., Liu Q.-F., Azim I., Zhu X., Yang J., Javed M.F., Rauf M. (2020). Prediction of mechanical properties of green concrete incorporating waste foundry sand based on gene expression programming. J. Hazard. Mater..

[52] Ferreira C. (2002). Gene expression programming in problem solving. Soft Computing and Industry.

[53] Shahmansouri A.A., Bengar H.A., Ghanbari S. (2020). Compressive strength prediction of eco-efficient ggbs-based geopolymer concrete using gep method. J. Build. Eng..

[54] Shah M.I., Javed M.F., Abunama T. (2020). Proposed formulation of surface water quality and modelling using gene expression, machine learning, and regression techniques. Environ. Sci. Pollut. Res..

[55] Khan M.I., Sutanto M.H., Khan K., Iqbal M., Napiah M.B., Zoorob S.E., Klemeš J.J., Bokhari A., Rafiq W. (2022). Effective use of recycled waste pet in cementitious grouts for developing sustainable semi-flexible pavement surfacing using artificial neural network. J. Clean. Prod..

[56] Salami B.A., Olayiwola T., Oyehan T.A., Raji I.A. (2021). Data-driven model for ternary-blend concrete compressive strength prediction using machine learning approach. Constr. Build. Mater..

[57] Farooq F., Ahmed W., Akbar A., Aslam F., Alyousef R. (2021). Predictive modeling for sustainable high-performance concrete from industrial wastes: A comparison and optimization of models using ensemble learners. J. Clean. Prod..

[58] Chou J.-S., Chong W.K., Bui D.-K. (2016). Nature-inspired metaheuristic regression system: Programming and implementation for civil engineering applications. J. Comput. Civ. Eng..

[59] Mohammed A., Burhan L., Ghafor K., Sarwar W., Mahmood W. (2021). Artificial neural network (ann), m5p-tree, and regression analyses to predict the early age compression strength of concrete modified with dbc-21 and vk-98 polymers. Neural Comput. Appl..

[60] Lübeck A., Gastaldini A., Barin D., Siqueira H. (2012). Compressive strength and electrical properties of concrete with white portland cement and blast-furnace slag. Cem. Concr. Compos..

[61] Shah H.A., Rehman S.K.U., Javed M.F., Iftikhar Y. (2021). Prediction of compressive and splitting tensile strength of concrete with fly ash by using gene expression programming. Struct. Concr..

[62] Donza H., Cabrera O., Irassar E. (2002). High-strength concrete with different fine aggregate. Cem. Concr. Res..

[63] Abdullahi M. (2012). Effect of aggregate type on compressive strength of concrete. Int. J. Civ. Struct. Eng..

[64] Beshr H., Almusallam A., Maslehuddin M. (2003). Effect of coarse aggregate quality on the mechanical properties of high strength concrete. Constr. Build. Mater..

[65] Wu K.-R., Chen B., Yao W., Zhang D. (2001). Effect of coarse aggregate type on mechanical properties of high-performance concrete. Cem. Concr. Res..

